# *Acanthodasys paurocactus* sp. n., a new species of Thaumastodermatidae (Gastrotricha, Macrodasyida) with multiple scale types from Capron Shoal, Florida

**DOI:** 10.3897/zookeys.190.2975

**Published:** 2012-05-04

**Authors:** Sarah Atherton, Rick Hochberg

**Affiliations:** 1 University of Massachusetts Lowell, One University Avenue, Lowell, MA , 01854, USA

**Keywords:** Meiofauna, Caribbean, gastrotrich, taxonomy, Macrodasyida, cuticle

## Abstract

A new species of *Acanthodasys* (Gastrotricha, Macrodasyida, Thaumastodermatidae) is described from sublittoral sediments off the Atlantic coast of Florida. *Acanthodasys paurocactus* **sp. n.** is a relatively small species (to 450 µm long) with a strap-shaped outline, a series of anterior, lateral, and ventrolateral adhesive tubes, paired caudal pedicles with posterior adhesive tubes, and a morphologically diverse cuticle. The cuticle contains both spined and unspined scales. Unspined scales are present in two general shapes: lanceolate and eye shaped, with some transitional shapes. All scales have a thickened rim and depressed central region; some scales of both shapes bear either one or more central bumps, a parallel ridge, or a perpendicular ridge that gives the appearance of a cross-shaped pattern under transmitted light. Spined scales are somewhat quadrangular in shape and bear uniancres to 15 µm long with a cross-shaped sectional profile. The new species is now one of five described species to possess both spined and spineless scales, and only one of two species to possess two types of spineless scales (the second species is an incompletely described specimen from Norway).

## Introduction

Gastrotrichs are microscopic invertebrates found in all oceans, seas and inland water bodies. The phylum Gastrotricha is composed of two orders, Chaetonotida, which includes 322 freshwater species (Balsamo et al. 2009) and 133 marine or brackish water species (Hummon and Todaro 2010), and Macrodasyida, which includes 295 marine species and 2 freshwater species (Hummon and Todaro 2010). As permanent members of the meiobenthos, marine gastrotrichs are often numerous in fine to medium grain sediments, ranking second or third in abundance behind nematodes and copepods (Todaro et al. 1995; Hochberg 1999). However, their minuscule size combined with a lack of taxonomic expertise across the globe has hindered studies of gastrotrich biodiversity, particularly in tropical regions like the Caribbean where even general studies of meiofauna are sorely lacking (Miloslavich et al. 2010).

To date, little is known of gastrotrich biodiversity in the tropics and subtropics, particularly the Tropical Northwestern Atlantic (TNWA, aka wider Caribbean), which extends from South Florida to the French Guiana-Brazil border. Todaro (1994) and Todaro et al. (1995) conducted the first surveys of gastrotrich biodiversity in the Gulf of Mexico, one of the five ecoregions that defines the TNWA, uncovering 45 species from Texas to the Florida peninsula. Other ecoregions have received attention by researchers looking to catalog their marine biodiversity including: the Bahamanian ecoregion (Renaud-Debyser 1963), the Central Caribbean ecoregion (Hummon 1974, 2010; Hochberg 2008, 2010; Hochberg and Atherton 2011), the Lesser Antilles ecoregion (Kisielewski 1984; Hummon 2010; Kånneby et al. 2012; Todaro et al. 2012) and the South Florida ecoregion (Thane-Fenchel 1970; Schöpfer-Sterrer 1974; Decho et al. 1985; Evans and Hummon 1991; Evans 1992, 1994; Hummon 2010). Hummon’s (2010) study remains the most extensive exploration to date, revealing species with distributions that span multiple ecoregions, thereby providing new insights into the biogeography of tropical and subtropical marine gastrotrichs.

In this study, we document a new species of *Acanthodasys* (Macrodasyida, Thaumastodermatidae) from sublittoral sediments off the Atlantic coast of Florida. This description forms part of a larger study that aims to classify the meiofauna from Capron Shoal, an offshore sandy shoal known to harbor diverse meiofauna (Winston and Håkansson 1986).

## Methods

Gastrotrichs were collected from Capron Shoal (27°26'52"N, 80°13'81"W), a 3 m deep station approximately 7 km off the coast of Fort Pierce, Florida. Samples were collected via anchor dredge in March, 2005 and August 2011 and analyzed back at the Smithsonian Marine Station in Fort Pierce, Florida. Extraction of gastrotrichs followed a standard protocol: 1) approximately 100 cm^3^ of sediment was combined with 900 cm^3^ of 7% aqueous MgCl2 solution in a 1 L Erlenmeyer flask and allowed to rest for 10 min; 2) the flask was gently shaken and the supernatant was decanted over a 48 µm mesh; and 3) the mesh was gently washed with seawater into a Petri dish. Specimens were sorted under a Leica EZ4 stereomicroscope, transferred to a glass slide, and viewed with a compound microscope (Zeiss A1) equipped with DIC (differential interference contrast). Light micrographs and digital videos were captured with a Sony Handycam digital camera. Measurements of individual specimens were performed with an ocular micrometer. Lengths and positions of organ systems are described in terms of percentage body units, where total body length from anterior (U00) to posterior (U100) is 100 units.

Specimens were prepared for scanning electron microscopy with the following protocol: fixation in 3% glutaraldehyde in 0.1M cacodylate buffer (pH 7.2) for 24h; rinsing four times (15 min each); postfixation in 1% OsO_4_ in 0.1 M cacodylate buffer for 1 h; rinsing in 0.1M cacodylate buffer (4 × 15 m); dehydration in an ethanol series; transfering to BEEM capsules and dehydration in a critical point dryer. Specimens were then sputter coated with gold and examined on a JEOL 6400 SEM at 10 kV.

One specimen was prepared for museum archival using the following protocol, which is deemed more permanent than standard glycerin mounts: fixation in 2.5% glutaraldehyde in 0.1M phosphate buffer saline (PBS; pH 7.4) for 24 hr; rinsing with PBS for 1 hr; postfixation in 1% OsO4 in 0.1M PBS for 30 sec (to increase contrast); rinsing in PBS for 15 min; dehydration through an ethanol series; transfering to propylene oxide for 30 min; and embedding in epon resin on a glass microscope slide (coverslipped and placed in an oven at 60^o^ C for 24 hr). Type specimen is deposited in the National Museum of Natural History, Smithsonian Institution, Washington, DC.

Abbreviations: PIJ, pharyngeointestinal junction; TbA, anterior adhesive tubes below ventral mouth rim; TbL, lateral adhesive tubes; TbP, posterior adhesive tubes on caudal pedicles; TbVl, ventrolateral adhesive tubes.

## Results

### Order Macrodasyida Remane, 1925 [Rao and Clausen, 1970]

Family Thaumastodermatidae Remane, 1927

Subfamily Diplodasyinae Ruppert, 1978

Genus *Acanthodasys* Remane, 1927

#### Acanthodasys paurocactus sp. n.

urn:lsid:zoobank.org:act:16C6323A-A944-4C69-9FBE-3F060876360F

http://species-id.net/wiki/Acanthodasys_paurocactus

##### Type locality.

Capron Shoal, Florida (27°26'52"N, 80°13'81"W), 3m depth, coarse sand. Sediments collected via anchor dredge by Hugh Reichardt and Woody Lee in March 2005; also in August 2011.

##### Materials examined.

Florida: Five adult specimens observed with DIC optics on 4 August 2011; two specimens prepared during an earlier expedition (March 2005) for scanning electron miocroscopy.

##### Holotype.

Adult specimen, ~ 375 µm long, curled, lateral orientation. Epidermal glands are artifically swollen. Cat no. USNM 1179053. Also, digital video of same specimen, live, deposited at the Smithsonian.

##### Diagnosis.

*Acanthodasys* with body length 300–450 µm (mature specimens at ~ 325 µm length). Body mostly strap-shaped with a distinct pair of caudal pedicles curled under body. Maximum body width at mouth/PIJ/midpoint of body is 35/42/67 µm. Pharynx to 136 µm long with pharyngeal pores near base. Area around mouth naked (no scales or spines) and up to 12 µm long, bearing numerous sensory cilia to 10 µm long. Scales cover entire body with oblique and transverse orientations; scales of two shapes, elongate lanceolate and short eye shaped, each with a centrally depressed region. Some scales have a small bump(s) or ridge at the center. Spined scales of dorsal and lateral cuticle bear uniancres 4–15 µm long; ventral uniancres 2–4 µm long scattered in ciliary fields and in median columns between locomotory cilia. Scales extend on to the caudal pedicles. Lateral sensory cilia to 15 µm long. Epidermal glands to 13 µm in diameter, 15–20 per side. Five TbA per side inserting directly on body surface at mouth rim. Up to 4 robust and elongate TbL per side, present only in trunk region. Up to 20 TbVl per side beginning posterior of PIJ, with the most posterior group of five TbVl becoming distinctly lateral in position close to the caudal pedicles. Caudal pedicles distinct with one lateral, two terminal, and one medial tube per lobe. Hermaphroditic, with paired testes and single glandular caudal organ. Rosette gland on dorsolateral left side of body; large egg present (~50 µm diameter); ovaries paired at caudal end.

##### Etymology.

This species is named for its spiky appearance, reminiscent of cactus (*pauro*, Greek: little, small; *cactus*, Greek: a prickly plant).

##### Description.

**Figure 1.**
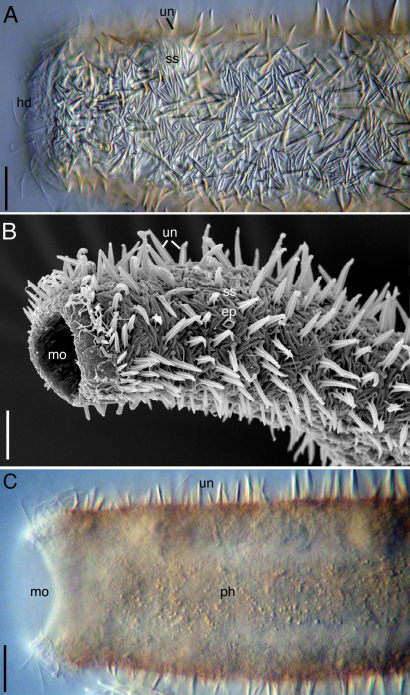
*Acanthodasys paurocactus* sp. n. **A** Adult specimen, dorsolaterally curled, DIC optics. Scale bar = 30 µm. **B** Adult specimen, ventral view, SEM. Note that the caudal pedicles are curled thereby obscuring the TbP. Scale bar = 50 µm. InsetPosterior end showing arrangement of TbVl and TbP. Scale bar = 14 µm. Abbreviatons: **ep** epidermal gland **oh** oral hood **lc** locomotory cilia **mo** mouth **TbL** lateral adhesive tube **un** uniancres.

**Figure 2.**
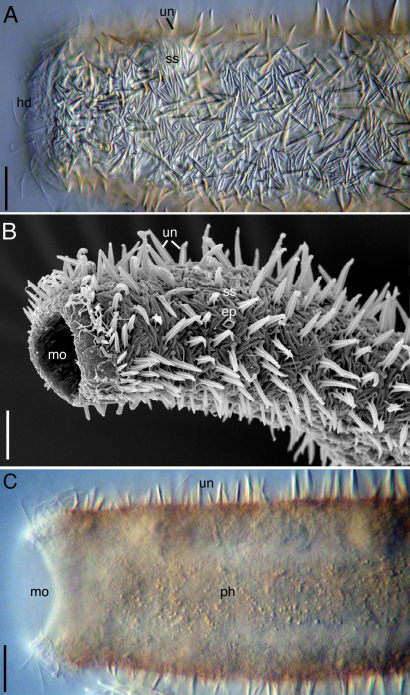
*Acanthodasys paurocactus* sp. n. **A** Closeup of anterior end of adult specimen, dorsal view, DIC optics. Scale bar = 15 µm. **B** Closeup of anterior end of adult specimen, lateral view, SEM. Scale bar = 15 µm. **C** Closeup of anterior end of adult specimen, ventral view, DIC optics. Scale bar = 12 µm. Abbreviations: **ep** opening of epidermal gland **hd** oral hood **mo** mouth **ph** pharynx **ss** spineless scales **un** uniancres.

The description is based on specimens measured *in vivo*; most specimens were dorsoventrally curled (see Fig. 1B). Body strap-shaped and 300–450 µm long (subadults ~ 300 µm long, most specimens 350–400 µm long) (Fig. 1). Terminal mouth 30–35 µm wide; body width increasing slightly to 43 µm at PIJ and to 67 µm in adults with developing ova. The trunk gradually tapers and leads to a pair of distinct caudal pedicles (Fig 1. inset). The entire body is covered with scales and spined scales except for the hood-like region around the mouth (Fig 1, 4). Epidermal glands to 13 µm diameter, up to 15–20 per side (Figs. 1A, 2).

**Figure 3.**
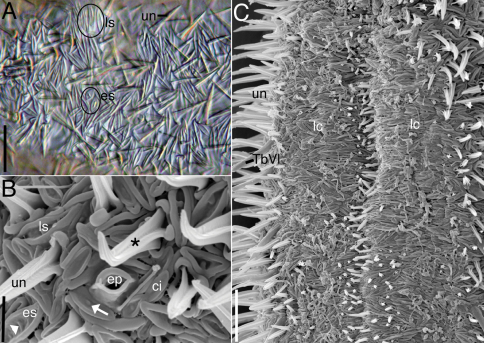
*Acanthodasys paurocactus* sp. n. **A** Closeup of dorsal cuticle of adult specimen, with focus on two types of spineless scales (circled) and uniancres (un), DIC optics. Scale bar = 12 µm. **B** Closeup of lateral cuticle of specimen showing lanceolate-shaped scales and eye-shaped scales, SEM. Some scales have perpendicular ridges (white arrowhead) or bumps (white arrow). Uniancres (*) arise from quadrangular-shaped scales. Scale bar = 4 µm. **C** Closeup of ventral trunk region of adult specimen showing location of ventral locomotory cilia (**lc**) and small ventral uniancres. Scale bar = 12 µm. Abbreviations: **ci** sensory cilium next to scales **ep** epidermal gland opening with raised cuticular ridge **es** eye-shaped scales **lc** locomotory cilia **ls** lanceolate scales **TbVl** ventrolateral adhesive tube **un** uniancre.

**Figure 4.**
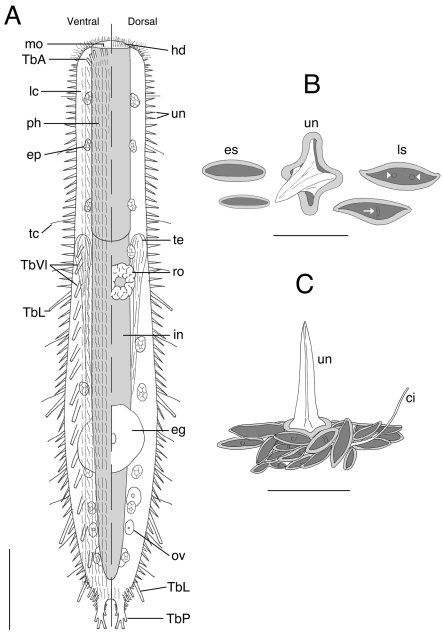
*Acanthodasys paurocactus* sp. n. **A** Composite sketch showing ventral (left) and dorsal (right) features. Scale bar = 40 µm. **B** Sketches of some scales based on SEM photographs of the dorsal cuticle. **C** Sketch of the general orientation of various spineless scales around a single spined scale based on SEM micrographs. Scale bar = 12 µm. Abbreviations: **ci** sensory cilium **eg** mature egg **ep** epidermal gland **es** eye-shaped scale **hd** oral hood **in** intestine **lc** locomotory cilia **ls** lanceolate-shaped scale **mo** mouth **ov** developing ova **ph** pharynx **ro** rosette organ **TbA** anterior adhesive tubes **TbL** lateral adhesive tubes **TbVl** ventrolateral adhesive tubes **TbP** posterior adhesive tubes **tc** lateral tactile cilia **te** testis **un** uniancres.

**Cuticlular armature.**Scales and spined scales present (Figs. 1A, 2–4). Scales often appear as interwoven fibers in brightfield optics, but DIC reveals numerous scales in between the spined scales (uniancres); several scales with various raised structures at their center (es, Fig. 3A). At high magnification with DIC (1000X) and SEM (> 1000X), at least two types of scales are observed: elongate, lanceolate-shaped scales (ls) and shorter, eye-shaped scales (es, Figs. 3, 4B); scales of intermediate size and shape are also present (Fig. 4B). All scales have a slightly thickened rim and central depression that extends along the longitudinal axis of the scale (Figs. 3B, 4B). Scales are arranged in several different orientations (longitudinal, transverse, oblique) across the dorsal and lateral body walls (Fig. 4C). SEM reveals that several scales, both lanceolate and eye shaped, have either a raised, oval bump at the center of the depression (white arrow, Fig. 3B) or a raised, bar-shaped ridge that is parallel (es, Fig. 3A) or perpendicular (white arrow, Fig. 3B) to the long axis of the scale. Lanceolate scales measure to 7 µm long and eye-shaped scales to 4 µm long with a maximum width to 1.5 µm. Spined scales bearing uniancres are interspersed among spineless scales (Fig. 3A–C). Uniancres with a cross-shaped (cruciform) sectional profile (asterisk, Fig. 3B) arise from the center of thick-rimmed scales that also have a somewhat quadrangular shape (Figs. 3B, 4B). Dorsal and lateral uniancres close to the oral hood are 3–5 µm long and increase in length along the trunk and reach a maximum of 15 µm long. Several small uniancres (2–3 µm) extend onto the caudal pedicles. Uniancres are mostly straight and oriented perpendicular to the body surface or in a slightly posterior direction; some uniancres had a bent tip that might have been the result of dehydration during preparation for SEM. Openings to the epidermal glands were surrounded by a raised cuticular ridge. Ventrolaterally, the uniancres decrease in size to 4 µm long where they border the locomotory cilia (Fig. 3C). Several very small uniancres, 1–3 µm long, are scattered among the cilia on the ventral body wall (Fig. 3C). Several tiny (1–2 µm) and slightly larger (2–4 µm) uniancres are present in between the ciliary columns in the trunk region.

**Cilia.**Sensory cilia to 10 µm long extend across the oral hood and form a thin corona around the head (Figs. 1A, 2A). A thicker patch of sensory cilia on either side of the head extends to 15 µm length. Smaller cilia 5–8 µm long line the mouth rim on the ventral body wall. At least ten stiff, hair-like cilia to 12 µm long extend down the length of the body on either side. Sensory cilia were observed to project out between the scales under SEM (Fig. 3B). Ventral locomotory cilia cover most of the pharyngeal region, extending from approximately U05 to the PIJ (Figs. 3C, 4A). At the PIJ, the cilia continues as a series of continuous rows to the posterior end but with a narrow column of naked cuticle (and uniancres) in between (Fig. 3C).

**Adhesive tubes.**Five pairs of anterior adhesive tubes (TbA) up to 5 µm long are present at the mouth margin: one either side of the midline is a close-set pair of tubes that is present medially and three tubes that form a group that is oriented diagnonally and closer to the lateral margin of the body (Fig. 4). Four pairs of TbL are present in the trunk region. Each tube is 21–25 µm long and robust in appearance. One specimen showed tubes at U45, U54, U70 and U80; three specimens were curled and difficult to measure. One specimen only had two TbL at positions U68 and U79. Up to twenty ventrolateral adhesive tubes (TbVl) to 12 µm long are inserted posterior to the PIJ. Most TbVl appear evenly spaced down the trunk; five TbVl become slightly more lateral in position and are clustered anterior to the caudal pedicles. The pedicles reach a maximum of 16 µm long including the posterior adhesive tubes (TbP) and bear a total of four TbP each: one lateral (6 µm), two terminal (4–5 µm), and one medial (4 µm) (Fig. 1 inset).

**Digestive tract.**Mouth terminal and circular to 35 um wide (Figs. 1B, 2B), surrounded by naked cuticle that forms a dorsal oral hood with a 12 µm rim (Fig. 2A, B); the naked cuticle around the ventral rim of the mouth is only 6 µm wide (hd, Figs. 2C). Pharynx to 136 µm long and 22 µm wide. Pharyngeal pores near base of pharynx (~ U34), not observable in all specimens. Intestine narrow and tapering at posterior; anus not observed.

**Reproductive system.**Hermaphroditic, with paired, bilateral testes beginning at the PIJ around U36 (Fig. 4A). Vasa deferentia extend posteriorly but could not be followed beyond mid-trunk region. Caudal organ observed in one specimen (body length: 400 µm), and pear-shaped, but the animal was too damaged for measurements. Rosette organ to 28 µm in diameter at U43–U46 in largest specimen (Fig. 4A). Paired ova were observed on either side of the posterior intestine in one specimem, with one large egg dorsal to intestine at approximately U65.

## Taxonomic remarks

At present, there are sixteen species of *Acanthodasys* known from several oceans and inland seas including the Atlantic ocean (e.g., Forneris 1961; Kisielewski 1987, Evans 1992), Indian ocean (e.g., Gerlach 1961; Naidu and Rao 2004), Black and Mediterranean seas (reviewed in Todaro et al. 2004), and other localities worldwide (reviewed in Hummon 2009). However, only nine species have formally published descriptions that meet the criteria of Article 13 of the ICZN (1999) including: *Acanthodasys aculeatus* Remane, 1927; *Acanthodasys algarvensis* Hummon, 2008 (see Hummon and Todaro 2010); *Acanthodasys arcassonensis* Kisielewski, 1987; *Acanthodasys caribbeanensis* Hochberg & Atherton, 2010; *Acanthodasys carolinensis* Hummon, 2008; *Acanthodasys fibrosus* Clausen, 2004; *Acanthodasys flabellicaudus* Hummon & Todaro, 2009; *Acanthodasys lineatus* Clausen, 2000; and *Acanthodasys silvulus* Evans, 1992. Five species named by Ruppert (1978) – *Acanthodasys diplodasyoides*, 1978, *Acanthodasys platydasyoides*, 1978, *Acanthodasys tetranchyrodermatoides*, 1978, *Acanthodasys thrinax*, 1978 and *Acanthodasys vermiformis*, 1978 – are considered *nomina nuda* according to Article 13 of the ICZN because they lack formal descriptions. Two species described from Norway (Clausen 2000) are incompletely known (*Acanthodasys* sp. 1, sp. 2) but presumably represent undescribed taxa. Of the described species, *Acanthodasys aculeatus* Remane, 1927 has the most extensive geographic distribution (but see below), and is only one of two species reported from the Tropical Northwestern Atlantic (Hummon 2009), the other species being *Acanthodasys caribbeanensis* Hochberg & Atherton, 2010 originally described from Panama in the Central Caribbean ecoregion.

In general, *Acanthodasys paurocactus* sp. n. can be easily distinguished from its congeners by the structure of the cuticle, while most other characteristics overlap with those of previously described species. For example, the strap-shaped body outline is characteristic of most species in the genus, while the presence of a pair of distinct caudal pedicles (lobes) is known from *Acanthodasys aculeatus*, *Acanthodasys carolinensis*, *Acanthodasys caribbeanensis*, *Acanthodasys fibrosus*, *Acanthodasys lineatus* and *Acanthodasys* sp. 1. Among these species, *Acanthodasys paurocactus* sp. n. shows the most overall similarity with *Acanthodasys aculeatus* sp. n. regarding body shape and general distribution of TbVl and TbP. Unfortunately, details about the number and distribution of adhesive tubes in *Acanthodasys aculeatus* are questionable as the original description by Remane is incomplete: “Die Verteilung der Haftröhrchen konnte ich nur teilweise feststellen” (1927: 213). Furthermore, recent accounts of *Acanthodasys aculeatus* from around the globe (e.g., Todaro et al. 1992; Fregni et al. 1999) indicate high variability in both body length and the number and position of adhesive tubes (e.g., Todaro et al. (1992) found TbD on specimens from the Tuscan archipelago that were not reported previously), calling into question the monophyletic status of *Acanthodasys aculeatus*. Therefore, the taxonomic status of *Acanthodasys aculeatus* must remain questionable until such time that further details, including genetic analyses, are provided on morphotypes from a wide range of geographic localities (e.g., see Kieneke et al. 2012 for genetic analyses of morphotypes of species of *Turbanella* across Europe).

*Acanthodasys paurocactus* sp. n. is now one of five species that is known to possess both spined scales (uniancres) and spineless scales. The other species are *Acanthodasys aculeatus*, *Acanthodasys arcassonensis*, *Acanthodasys caribbeanensis* and *Acanthodasys* sp. 2. The uniancres of the new species are larger than those reported for *Acanthodasys aculeatus* (variable: up to 9 µm, Forneris 1961), *Acanthodasys arcassonensis* (11 µm), and *Acanthodasys* sp. 2 (6–10 µm), but much smaller than those of *Acanthodasys caribbeanenesis* (up to 50 µm). Interestingly, the zone between spined scales appears to be made of a series of fine, interwoven cuticular fibers (based on transmitted light) similar to that of *Acanthodasys fibrosus* Clausen, 2004, which lacks spineless scales. Closer inspection with differential interference contrast (DIC) at high magnification, however, revealed two important characteristics: all uniancres arise from a scale that makes the entire structure appear as an inverted t-shape (similar to that observed for *Acanthodasys flabellicaudus*), and there are numerous spineless scales in between the uniancres. However, only when specimens were examined with scanning electron microscopy (SEM) could we unambiguously discern the structure of the spineless scales.

There were two general types of spineless scales revealed with SEM. One type of scale was lanceolate in shape, very thin and up to 7 µm long. Interspersed among these scales were eye-shaped scales that were somewhat wider and to 4 µm long. Scales of intermediate size and shape were also present. All scales had a central depressed region that extended the length of the longitudinal axis of the scale; some of these scales also had raised regions (e.g., bumps, a single parallel ridge, a single perpendicular ridge) in the depressed region. The rim of all scales, which appeared thicker than than the rest of the scale body, was always elevated above the central depression. We hypothesize that the raised ridge and depressed center of each scales alters their refractive index under transmitted light, thereby imparting the fiber-like appearance of the scales at low magnification. A similar case may also be found in *Acanthodasys fibrosus* once that species is viewed with SEM. Interestingly, *Acanthodasys paurocactus* sp. n. is now only the second known species to possess two types of spineless scales, the other species being an undescribed specimen (*Acanthodasys* sp. 2) from Norway (Clausen 2000). Unfortunately, many details on the undescribed species remain to be determined, but based on Clausen’s measurements of the spines (6–10 µm) and body length (800 µm), his specimens are clearly different from those present in Florida.

As noted for *Acanthodasys arcassonensis* (Kisielewski 1987) and other species of *Acanthodasys* (Rieger and Rieger 1977), the uniancres of *Acanthodasys paurocactus* sp. n. have a cross-shaped or cruciform sectional profile under transmitted light. This profile is reminiscent of the patterns noted in various spineless scales of the new species and spineless scales of species of *Diplodasys* (Rieger and Rieger 1977). We hypothesize that this pattern may be due to the presence of a perpendicular ridge in the center of some scales (see Fig. 3B, white arrowhead), which at low magnifications and under transmitted light can make the scale appear to have a cross-like pattern. Rieger and Rieger (1977) made similar observations on species at the ultrastructural level, and hypothesized that the cross-shape pattern characteristic of these scales (and the uniancres) may be plesiomorphic within the subfamily Diplodasyinae. Further observations on other species with SEM are warranted before accepting the cross-shaped pattern as a useful taxonomic character.

## Supplementary Material

XML Treatment for Acanthodasys paurocactus
